# Assessment of a Comparative Bayesian-Enhanced Population-Based Decision Model for COVID-19 Critical Care Prediction in the Dominican Republic Social Security Affiliates

**DOI:** 10.7759/cureus.26781

**Published:** 2022-07-12

**Authors:** Amado A Baez, Oscar J Lopez, Maria Martinez, Colyn White, Pedro Ramirez-Slaibe, Leticia Martinez, Pedro L Castellanos

**Affiliations:** 1 Emergency Medicine/Public Health/Epidemiology, Augusta University/Medical College of Georgia, Augusta, USA; 2 Postgraduate Studies, Universidad Nacional Pedro Henríquez Ureña, Santo Domingo, DOM; 3 Internal Medicine Residency, University of Texas Rio Grande Valley, Rio Grande, USA; 4 Surgery, University of Washington Medical Center, Washington, USA; 5 Health Sciences, University of South Florida, Tampa, USA; 6 Health Sciences, Centro de Investigación y Estudios en Gerencia de Salud, Santo Domingo, DOM; 7 Actuarial, Superintendencia de Salud, Santo Domingo, DOM; 8 Epidemiology and Public Health, Superintendent of Health and Labor Risks (SISALRIL), Santo Domingo, DOM

**Keywords:** curb-65, national early warning score, icu, intensive care, risk, population-based, covid-19

## Abstract

Introduction: The novel coronavirus disease 2019 (COVID-19) has been a major health concern worldwide. This study aims to develop a Bayesian model to predict critical outcomes in patients with COVID-19.

Methods: Sensitivity and specificity were obtained from previous meta-analysis studies. The complex vulnerability index (IVC-COV2 index for its abbreviation in Spanish) was used to set the pretest probability. Likelihood ratios were integrated into a Fagan nomogram for posttest probabilities, and IVC-COV2 + National Early Warning Score (NEWS) values and CURB-65 scores were generated. Absolute and relative diagnostic gains (RDGs) were calculated based on pretest and posttest differences.

Results: The IVC-COV2 index was derived from a population of 1,055,746 individuals and was based on mortality in high-risk (71.97%), intermediate-risk (26.11%), and low-risk (1.91%) groups. The integration of models in which IVC-COV2 intermediate + NEWS ≥ 5 and CURB-65 > 2 led to a "number needed to (NNT) diagnose" that was slightly improved in the CURB-65 model (2 vs. 3). A comparison of diagnostic gains revealed that neither the positive likelihood ratio (P = 0.62) nor the negative likelihood ratio (P = 0.95) differed significantly between the IVC-COV2 NEWS model and the CURB-65 model.

Conclusion: According to the proposed mathematical model, the combination of the IVC-COV2 intermediate score and NEWS or CURB-65 score yields superior results and a greater predictive value for the severity of illness. To the best of our knowledge, this is the first population-based/mathematical model developed for use in COVID-19 critical care decision-making.

## Introduction

On January 30, 2020, the World Health Organization declared the novel coronavirus disease 2019 (COVID-19), caused by the severe acute respiratory syndrome coronavirus-2 (SARS-CoV-2), as a public health emergency [1]. Respiratory failure is the leading cause of mortality in patients with COVID-19 [2]; myocardial injury, kidney or liver injury, and multiorgan dysfunction are among other complications that may lead to death [3]. Several prognostic factors, including older age, male gender, comorbidities, and smoking, have been associated with severe disease or death [4-7].

The National Institute for Health and Care Excellence recommends the use of the National Early Warning Score (NEWS) in critical care in its guidelines on the management of COVID-19 [8,9]. NEWS is a standardized clinical scoring system that has been developed to enhance the detection of deterioration in acute patients. It is based on a logistic regression model that predicts in-hospital patient mortality within 24 hours of observing specific vital signs [10] that originally included pulse rate, respiratory rate, blood pressure, temperature, and oxygen saturation. NEWS2 is the latest version of NEWS, adding onset of confusion as one of the parameters, as well as adding 2 points for patients requiring supplemental oxygen to maintain their recommended oxygen saturation level. In-hospital studies have shown that NEWS2 did not add any predictive value over that of NEWS even in patients with type 2 respiratory failure [11-14]. Therefore, for this model, we integrated the sensitivities and specificities of the original NEWS.

Other scoring systems, such as CURB-65, have been widely used to predict the 30-day mortality rate in patients with community-acquired pneumonia [15]. CURB-65 was useful in predicting 14-day mortality in patients with hospital-acquired pneumonia [16]. In addition, CURB-65 was integrated to develop a simple predictive tool for estimating the risk of 30-day mortality and stratifying patients with COVID-19 [17].

The complex vulnerability index (abbreviated in Spanish as IVC-COV2) [18] is a population-based index designed by the Dominican Republic Health and Risk Superintendence in 2020 to identify individuals at risk of COVID-19. This index considers sex, age, and comorbidities to assess the risk of a patient with COVID-19 experiencing a critical outcome. Patients in the present study were stratified as low, intermediate, and high-risk based on their IVC-COV2 score. In general, the use of such clinical scoring systems to predict severe disease and mortality in patients with COVID-19 should be investigated further in larger prospective studies.

Bayesian statistics have been used with mathematical probability instruments to evaluate uncertainty. Our group has been studying Bayesian statistics in clinical/medical decision-making and as a "data recycling" tool; such statistics can be used to compare the diagnostic quality of different serum biomarkers using a methodology that calculates the probability of an event based on criteria related to the specific event [19-26]. Our group developed a simple mathematical method, known as "Bayesian Diagnostic Gains" (BDGs), for interpreting the diagnostic impact. In this method, the relative diagnostic gain (RDG) and absolute diagnostic gain (ADG) are calculated based on the differences deducted from pre- and posttest probabilities as follows: ADG = posttest − pretest; RDG = 100 × posttest − pretest/pretest. This is our first attempt to integrate BDGs into a critical care prediction multi-item model for COVID-19.

The objective of this study is to develop a hybrid mathematical model to assist in predicting critical care disposition in patients with COVID-19 using Bayesian statistics and a comparative assessment of the IVC-COV2 score integrated with the NEWS and CURB-65 systems.

This article was published as a temporary preprint at https://www.researchsquare.com/article/rs-991050/v1 on October 19, 2021.

## Materials and methods

Calculations for sensitivity and specificity were obtained from previous meta-analysis studies [11,15]. The IVC-COV2 index [18] was developed on the basis of a captive insured population and derived by recursive partitioning from four dimensions (Table 1), namely, (1) comorbidities, (2) age, (3) gender, and (4) social/family (Tables 2-3), using the following formula: IVC-COV2 = .3𝐷1 +.3𝐷2 +.3𝐷3 +.1𝐷4.

**Table 1:** IVC-COV2 COVID-19 index dimensions.

Dimension #1 (D1)	Dimension #2 (D2)	Dimension #3 (D3)	Dimension #4 (D4)
Comorbidities	Age	Gender	Family/social
Index construction: IVCCoV2=.3D1 +.3D2 +.3D3 +.1D4			

**Table 2:** Population-based dimension #1 (D1) assessment of risk factors associated with death.

Risk factor	Percentage (N = 157)
Diabetes	33.12%
Hypertension	61.15%
Emphysema	15.92%
Peripheral vascular disease	1.91%
Cancer	14.64%
Chronic renal failure	13.38%
Cardiovascular disease	16.56%

**Table 3:** IVC-COV2 index population assessment.

IVC-COV2 risk level	Vulnerable population (count)	Alive (count)	Deceased (count)	Percentage of mortality according to IVC-COV2 risk
Total	1,055,745	1,055,588	157	100%
Low	393,459	393,456	3	1.91%
Intermediate	395,172	395,131	41	26.11%
High	267,001	267,001	113	71.97%

We used the IVC-COV2 index to set the pretest probability, calculated the likelihood ratios, and integrated these into a Bayesian/Fagan nomogram to attain posttest probabilities, thereby generating a sequential value for IVC-COV2 + NEWS.

To quantify the diagnostic impact, we developed a BDG framework wherein RDG and ADG were calculated based on the differences between the pretest results and posttest probabilities of CURB-65 (Table 4) and NEWS-2 (Table 5). ADG and RDG were calculated as previously described in this study (Table 6).

**Table 4:** The CURB-65 severity score.

Method: Score 1 point for eachof the following features that are present	Confusion (mental testscore 8 new disorientation in person, place, or time)
	BUN > 20 mg/dL
	Respiratory rate 30 breaths/min
	Blood pressure (systolic<90 mm Hg, or diastolic 60 mm Hg)
	Age 65 years
Interpretationof CURB-65 score	0-1: Probably suitable for home treatment;low risk of death
	2: Consider hospital-supervised treatment
	3:Manage in hospital as severe pneumonia; high risk of death

**Table 5:** The NEWS scoring system.

	Score						
Physiological parameter	3	2	1	0	1	2	3
Respiration rate (per minute)	≤8		9–11	12–20		21–24	≥25
SpO_2_ scale 1 (%)	≤91	92–93	94–95	≥96			
SpO_2_ scale 2 (%)	≤83	84–85	86–87	88–92 ≥93 on air	93–94 on oxygen	95–96 on oxygen	≥97 on oxygen
Air or oxygen?		Oxygen		Air			
Systolic blood pressure (mmHg)	≤90	91–100	101–110	111–219			≥220
Pulse (per minute)	≤40		41–50	51–90	91–110	111–130	≥131
Consciousness				Alert			CVPU
Temperature (°C)	≤35.0		35.1–36.0	36.1–38.0	38.1–39.0	≥39.1	

**Table 6:** NEWS and CURB-65 score sensitivity, specificity, and positive (+) and negative (−) likelihood ratios. LR (+): positive likelihood ratio LR (−): negative likelihood ratio.

	Sensitivity	Specificity	LR (+)	LR (−)
NEWS > 5	86.7%	90.5%	9.13	0.15
CURB-65	73.0 %	85.0 %	4.87	0.32

The number needed to (NNT) metric often has a more intuitive appeal to clinicians compared with standard diagnostic accuracy measures. Such metrics have been used to accurately treat, diagnose, and/or predict disease in certain populations. The NNT refers to the number of patients who must be treated to prevent one additional bad outcome. The NNT is the inverse of the absolute risk reduction (ARR), which is the absolute difference in the rates of events between a given activity or treatment relative to a control activity or treatment, i.e., the control event rate (CER) minus the experimental event rate (EER), or ARR = CER − EER. NNTs are always rounded up to the nearest whole number and are accompanied by 95% confidence intervals as standard. For example, if a drug reduces the risk of a bad outcome from 50% to 40%, then the ARR is calculated as ARR = 0.5 − EER.

Based on this concept, we used ADG to create a formula to calculate the number needed to diagnose (NND), which we termed the Bayesian NND (B-NND). To develop this formula, we used the statistical basis of the NNT formula but substituted ADG for ARR: NND = 1/ADG. Descriptive statistics with confidence intervals were used to represent group characteristics, and a t-test was used to establish the comparative differences between models; the statistical significance was set at P < 0.05.

## Results

The IVC-COV2 score was derived using four dimensions (Table 1) of a captive insured population of 1,055,745 Dominican social security affiliates. Table 2 presents the results of a comorbidity analysis, whereas Table 3 presents the risk analysis breakdown of the population, which shows that 393,459 (1.91%) patients were at low risk, 395,172 (26.11%) were at intermediate risk, and 267,114 (71.97%) were at high risk. The IVC-COV2 score was integrated into our Bayesian model, which comparatively included the NEWS and CURB-65 likelihood ratios to obtain the posttest probability. Table 4 presents the pooled sensitivity and specificity for NEWS and CURB-65 [11,15].

Table 7 presents the combination of low, intermediate, and high risk in the IVC-COV2 index with NEWS and CURB-65 low, intermediate, and high results. Using a low pretest probability (1.91%) associated with low IVC-COV2 with NEWS of ≥5, the positive posttest probability was 15% (95% CI: 13%-17%), whereas the negative posttest probability was 0% (95% CI: 0-1%). Using an intermediate pretest probability (26.11%) associated with intermediate IVC-COV2 with NEWS ≥5, the positive posttest probability was 76% (95% CI: 72-78%), whereas the negative posttest probability was 4% (95% CI: 3%-5%). Combining the high pretest probability (71.97%) of high IVC-COV2 with NEWS 2 of ≥5 yielded a positive posttest probability of 96% (95% CI: 93%-97%) and a negative posttest probability of 28% (95% CI: 26-29%).

**Table 7:** Bayesian modeling results. LR (+): positive likelihood ratio, LR (−): negative likelihood ratio, ADG: absolute diagnosis gain, NND: number needed to diagnose

	Pretest probability (%)	Posttest probability (%)	Absolute gain (%)
COVID-19 VI low + NEWS	1.91	LR (+) 15.0 LR (−) 0	LR (+) 13.09 LR (+) 1.91
COVID-19 VI intermediate + NEWS	26.11	LR (+) 76.0 LR (−) 4.0	LR (+) 49.89 LR (−) 22.11
COVID-19 VI high + NEWS	71.97	LR (+) 96.0 LR (+) 28.0	LR (+) 24.03 LR (+) 43.97
COVID-19 VI low + CURB-65 >2	1.91	LR (+) 9.00 LR (−) 1.00	LR (+) 7.09 LR (−) 0.91
COVID-19 VI intermediate +CURB-65 >2	26.11	LR (+) 63.00 LR (+) 10.00	LR (+) 36.89 LR (+) 16.11
COVID-19 VI high + CURB-65 >2	71.97	LR (+) 93.00 LR (−) 45.00	LR (+) 21.03 LR (−) 26.97

On assessing the models with CURB-65 > 2 (Table 5), the posttest probability scores for the positive likelihood ratios were as follows: low = 15% (95% CI: 12-19%), intermediate = 76% (95% CI: 72-80%), and high = 96% (95% CI: 94-97%). In contrast, the posttest probability scores for the negative likelihood ratios were as follows: low = 0% (95% CI: 0-1%), intermediate = 5% (95% CI: 4-7%), and high = 28% (24-32%).

Table 8 presents the B-NND analysis for both the IVC-CoV2 NEWS and CURB-65 models. A slight improvement was detected in the NEWS model, i.e., 2.00 (2) vs. 2.71 (3). A comparative assessment of ADG and RDG revealed no statistical differences between the IVC-CoV2 NEWS model and the CURB-65 model in terms of the positive likelihood ratio (P = 0.62) or negative likelihood ratio (P = 0.95).

**Table 8:** Bayesian numbers needed to diagnose LR (+): positive likelihood ratio; ADG: absolute diagnosis gain; NND: number needed to diagnose

Pretest probability (intermediate only)	Posttest LR (+)	ADG (%)	NND (rounded)
COVID-19 VI (26.11)	NEWS 58	49.89	2.00 (2)
COVID-19 VI (26.11)	CURB 65-63	36.89	2.71 (3)

## Discussion

Mathematical modeling offers potential solutions for probability and uncertainty in medicine. The concept of data recycling is traditionally applied in other statistical methodologies, such as meta-analysis. We propose using high-quality published data and integrating independent studies into a Bayesian model to find solutions to unresolved clinical problems and generate novel hypotheses. Our model could be a unique addition to the modeling toolbox.

Few studies have focused on the use of NEWS/NEWS2, particularly for patients with COVID-19, even in a hospital setting. A study conducted in China during the early phase of the COVID-19 pandemic suggested using an early warning score that was based on an adapted version of the NEWS2. In this system, age >65 years was given 3 points, which supported the fact that age is an independent risk factor for COVID-19 survival [11-13]. In another study, one of the four hospitalized patients with COVID-19 had severe disease, and the in-hospital mortality was 20% [13]. Using NEWS at the time of admission to the emergency department could predict severe disease and in-hospital mortality; NEWS is superior to qSOFA and other clinical risk scores for this purpose. In another study, the CURB-65 score was used to predict mortality associated with pneumonia and ICU disposition [15].

The biggest diagnostic gains in our models were found when integrating the NEWS and CURB-65 scores for the low and intermediate pretest probability subgroups. According to the IVC-COV2 index and NEWS, high-risk patients should be monitored more closely because of the higher risk of critical outcomes. In clinical practice, multiple factors, such as history, physical examination, laboratory results, and radiologic results, are used as a guide in clinical decision-making and unusually intermediate (moderate) scores in decision rules can lead to a lack of clarity and the need for enhancements. Rapidly producing data-driven results during public health emergencies is of utmost importance because it guides authorities and clinicians in decision-making using the immediately available evidence. The use of insurance population-based data combined with the existing decision-making rules, all packaged within a strong mathematical model, will be particularly useful in critical care decision-making and resource allocation.

The integration of the IVC-COV2 index with NEWS and CURB-65 provided an improved posttest probability in the rule-in as well as rule-out subgroups. Thus, the Bayesian/patient-centered clinical decision tool developed in this study could be used to integrate independent clinical items to adequately predict disease severity and eventual ICU resource utilization. Moreover, the proposed model containing these scores independently integrated with the IVC-COV2 index is expected to have improved predictive value for ICU and ward admissions for patients with COVID-19. This decision format is aligned with the realities of clinical practice wherein the specifics of one patient are added to the integrated value of various tests prior to taking diagnostic and/or therapeutic decisions. We believe that this approach will be especially helpful to guide decision-making in low-resource settings and assist clinical staff who are not physicians, particularly if it can be integrated as a computer-based decision tool.

The number needed to diagnose is generally defined as the number of patients who must be examined to correctly detect one person with the disease of interest in a study population including people with and without the known disease. In diagnostic testing, low NND values are desired (NND = 1 is close to perfect). This study expands on the NND concept by integrating a novel evidence-based tool, B-NND, which allows the clinical application of Bayesian statistics. We believe that it is a relatively simple tool for visualizing the impact of probability mathematics, especially compared with other mathematical models and their effectiveness. Based on our NND results and comparative analyses, the IVC-COV2 index with NEWS/CURB-65 is a better predictor of ICU disposition needs than the IVC-COV2 index alone. The B-NND score of 2 assigned to the integration of NEWS suggests a higher diagnostic value, although this was not detected in the t-test model. The low and intermediate IVC-COV2 index integrated with NEWS or CURB-65 was the best predictor of COVID-19 ICU needs.

In this study, we experimented with the concept of "data recycling," wherein data and results from different studies were integrated into one probability model to enable making rapid clinical decisions related to areas that have not been studied or to generate new hypotheses. Regarding COVID-19, similar population-based hybrid math models could potentially be used for vaccine campaign strategies. There are some limitations to the current study, such as the small sample size, data being taken from a single country, the intrinsic qualities of the mathematical model, and the absence of strong prospective studies to validate the use of NEWS and CURB-65 in COVID-19 care. Nevertheless, the use of clinical scoring systems to predict severe disease and mortality in patients with COVID-19 should be investigated further in larger prospective studies.

## Conclusions

According to our theoretical mathematical model, the combination of the IVC-COV2 intermediate index and NEWS or CURB-65 yielded superior predictive results compared with those using other probability settings, thus suggesting the improved predictive value of illness severity and admission level care needs. Furthermore, our results advocate the rapid deployment of decision support tools that can combine these various clinical items into a final pathway for the prediction of admissions in patients with COVID-19. The NEWS and CURB-65 models were not significantly different. Thus, we recommend institutions use the scoring system that best applies to their setting. To the best of our knowledge, this is the first hybrid population-based/mathematical model for COVID-19 critical care decision-making.
